# Leveraging Nanocrystal HKUST-1 in Mixed-Matrix Membranes for Ethylene/Ethane Separation

**DOI:** 10.3390/membranes10040074

**Published:** 2020-04-16

**Authors:** Chong Yang Chuah, S.A.S.C. Samarasinghe, Wen Li, Kunli Goh, Tae-Hyun Bae

**Affiliations:** 1 Singapore Membrane Technology Centre, Nanyang Environment and Water Research Institute, Nanyang Technological University, Singapore 637141, Singapore; chongyang.chuah@ntu.edu.sg (C.Y.C.);; 2 School of Chemical and Biomedical Engineering, Nanyang Technological University, Singapore 637459, Singapore; liwe0025@e.ntu.edu.sg; 3 Department of Chemical and Biomolecular Engineering, Korea Advanced Institute of Science and Technology, Daejeon 34141, Korea

**Keywords:** C_2_H_4_/C_2_H_6_ separation, HKUST-1, gas permeation, solubility, diffusivity

## Abstract

The energy-intensive ethylene/ethane separation process is a key challenge to the petrochemical industry. HKUST-1, a metal–organic framework (MOF) which possesses high accessible surface area and porosity, is utilized in mixed-matrix membrane fabrication to investigate its potential for improving the performance for C_2_H_4_/C_2_H_6_ separation. Prior to membrane fabrication and gas permeation analysis, nanocrystal HKUST-1 was first synthesized. This step is critical in order to ensure that defect-free mixed-matrix membranes can be formed. Then, polyimide-based polymers, ODPA-TMPDA and 6FDA-TMPDA, were chosen as the matrices. Our findings revealed that 20 wt% loading of HKUST-1 was capable of improving C_2_H_4_ permeability (155% for ODPA-TMPDA and 69% for 6FDA-TMPDA) without excessively sacrificing the C_2_H_4_/C_2_H_6_ selectivity. The C_2_H_4_ and C_2_H_6_ diffusivity, as well as solubility, were also improved substantially as compared to the pure polymeric membranes. Overall, our results edge near the upper bound, confirming the effectiveness of leveraging nanocrystal HKUST-1 filler for performance enhancements in mixed-matrix membranes for C_2_H_4_/C_2_H_6_ separation.

## 1. Introduction

Olefin is one of the most important building blocks in the petrochemical industry. One example is ethylene (C_2_H_4_). The global annual production of ethylene reached to almost 150 million tons in 2016, and is projected to exceed 200 million tons in the year of 2020 [1,2]. However, looking forward, the need for future energy security through renewable energies, the measurable socio-economic impact due to climate change, and the inexorable call for a circular economy, are likely to drive a transition in the petrochemical industry to generate value-creating opportunities to deal with the shrinking demand for ethylene, and an impending slower market growth [2,3]. Despite that, ethylene remains as an active pharmaceutical ingredient and is used in the production of polymers such as polyethylene and polyvinyl chloride [4,5,6]. Ethylene (alkene in general) is typically produced via hydrocarbon cracking (either from naphtha in crude oil or ethane from natural gas). There are also efforts in producing ethylene through environmental-friendly means, such as ethylene biosynthesis in biorefinery [7,8,9]. However, doing so requires a complex coalition of both the petrochemical and biorefining industries, which will involve challenges including finding affordable feedstock access, enabling capabilities for industrial-scale commercialization and value-chain integration, as well as soliciting stable and supportive regulations by the government [10,11]. Regardless of how the industry eventually evolves, light hydrocarbon separation, such as the separation of ethylene from its paraffin counterpart, ethane (C_2_H_6_), is deemed necessary after contaminants removal to give the precursor [12,13]. Essentially, ethylene/ethane (C_2_H_4_/C_2_H_6_) separation is critical as the purity of the ethylene precursor directly influences the quality of the high-value commercial end-products.

In a typical petrochemical process where high purity ethylene feed is desired, a high-pressure (c.a. 23 bar) cryogenic distillation process that operates at low temperature (−160 °C) is necessary to separate ethylene from ethane at the desired purity in view of their close melting and boiling points. This process is energy intensive, with 75–85% of the total cost expected to be siphoned out from the ethylene production [1,12,14,15,16,17]. Adsorptive separation via pressure swing adsorption (PSA) is also energy-intensive as it requires additional energy supply to regenerate the saturated adsorbents for enabling a repetitive adsorption–desorption cycling process. In comparison, gas separation membrane technology is comparatively low cost and energy efficient as the separation is carried out without any phase change and usually under ambient conditions [18,19,20,21,22,23]. Polymeric membranes are at the core of this technology, considering their high mechanical stability and ease of processability. Nonetheless, conventional polymeric membranes are hampered by an inevitable permeability–selectivity trade-off given that solution–diffusion is the main separation mechanism [24,25,26,27,28,29]. Apart from this, inorganic membranes, such as those based on pure zeolites and metal–organic frameworks (MOFs), are typically high-performing but hampered by their poor scalability potential [30]. Beyond membrane performances, other real-life challenges, such as effects of high temperatures and pressures, presence of moisture in the feed gas and impurities such as H_2_S that are damaging to the membranes, must be addressed to realize industrial applications [31,32,33,34,35,36,37]. However, in this work, our aim is to develop mixed-matrix membranes, which amalgamate the merits of both polymeric and inorganic membranes, to achieve enhanced C_2_H_4_/C_2_H_6_ separation.

Mixed-matrix membranes achieve enhanced separation performance of the polymeric matrices by incorporating nanoporous materials as fillers to provide pathways of higher diffusion coefficient to engineer the transport properties of the membranes [35,38,39,40]. To date, MOF is an attractive filler material for mixed-matrix membranes for gas separation process, owing to its large pore volume and accessible surface area for increasing gas diffusivity. On the other hand, facile post- and pre-synthetic functionalization of MOFs can enhance the affinity of the gas molecules to the filler materials, leading to an improvement in gas solubility [36,37,41,42]. More importantly, MOFs is more compatible with the organic polymer matrix in view of the moieties in the frameworks that are similarly organic in nature. This eliminates the need for compatibilizers to enhance the interfacial morphology between the polymer and filler [43,44].

For these reasons, we have chosen to focus on HKUST-1 to investigate its potential utility as a filler for mixed-matrix membrane for C_2_H_4_/C_2_H_6_ separation. HKUST-1 possesses large square pores of 9 × 9 Å and, more importantly, unsaturated metal sites that favor interaction towards the π-electron system (sp^2^ hybridized) of the ethylene molecules. In comparison with other well-reported MOFs, such as IRMOF-8, ZIF-7, and ZIF-8, which do not contain these strong metal sites, favorable interaction with C_2_H_4_ is improbable, resulting in less competitive C_2_H_4_/C_2_H_6_ separation [45,46,47]. Furthermore, as compared to MOF-74, which contains open metal sites, HKUST-1 is generally more tolerable towards water vapor and humidity, rendering it more practical for mixed-matrix membrane fabrication [48,49]. At present, micron-sized HKUST-1 is commercially available under the tradename Basolite C300 (produced by BASF, Ludwigshafen, Germany). Nonetheless, the particle size is too large for membrane fabrication. Hence, we chose to synthesis nanocrystals of HKUST-1 to increase its effectiveness as a nanoporous filler for the development of mixed-matrix membrane [50].

In this work, C_2_H_4_/C_2_H_6_ separation performance of mixed-matrix membranes is conducted using a 50/50 mixture gas. Nanocrystal HKUST-1 was incorporated into two glassy polymers, namely ODPA-TMPDA and 6FDA-TMPDA (The abbreviations of these polymers will be explained in Section 2.1). These polymers were selected for its higher C_2_H_4_ permeability as compared to commercially available glassy polymers (e.g., Matrimid^®^, polysulfone, and Ultem^®^/P84) [32,33,51,52]. Our results showed that, with the addition of HKUST-1 nanocrystals, a clear improvement in C_2_H_4_ permeability of the membranes can be observed. Comprehensive analyses (C_2_H_4_ and C_2_H_6_ solubility and diffusivity) on the addition of HKUST-1 nanocrystals in polymeric membrane were evaluated to elucidate the possible reasons behind the improved performances.

## 2. Materials and Methods

### 2.1. Materials

2,4,6-trimethyl-*m*-phenylenediamine (TMPDA), 4,4′-(hexafluoroisopropylidene)diphthalic anhydride (6FDA), 4,4′-oxydiphthalic anhydride (ODPA), acetic anhydride (Ac_2_O), copper(II) nitrate trihydrate (Cu(NO_3_)_2_.3H_2_O), *N*-methyl-2-pyrrolidone (NMP), triethylamine (TEA), and trimesic acid (C_9_H_6_O_6_) were purchased from Sigma Aldrich (Singapore). Absolute ethanol, dimethylacetamide (DMAc), methanol, and chloroform were purchased from VWR (Singapore). For the synthesis of 6FDA-TMPDA, the respective monomers were purified first via a sublimation process (under vacuum). All other chemicals and reagents were used as received.

### 2.2. Synthesis of Nanocrystal HKUST-1

Nanocrystal HKUST-1 were developed based on the method use in the literature as indicated elsewhere [53]. First, addition of Cu(NO_3_)_2_.3H_2_O (1.2 g) was conducted in a glass vial that contains absolute ethanol (20 mL). Next, C_9_H_6_O_6_ (0.6 g) was added and agitated at room temperature with relative humidity of 60–70% for 1 day. Vacuum filtration was used to recover the HKUST-1 nanocrystals under extensive washing by copious amounts of ethanol:water mixture in the volume ratio of 1:1.

### 2.3. Synthesis of ODPA-TMPDA Polymer

**Figure 1:**
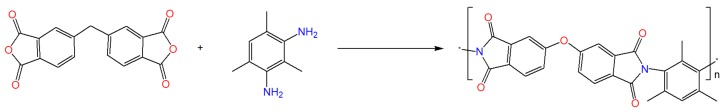
Reaction scheme of ODPA-TMPDA synthesis.

The ODPA-TMPDA polymer (Figure 1) was synthesized under an inert atmosphere based on the procedures as elaborated below [39,54]. First, TMPDA (1.63 g) was added into a flask, which was followed by the incorporation of DMAc (20.0 g). The solution was vigorously stirred before the addition of ODPA (3.36 g) into the resulting mixture. The mixture was then stirred for 1 day to obtain a viscous polyamic acid solution. Next, 4.39 g of TEA and 4.44 g of Ac_2_O were added to the solution to initiate an imidization process. The solution was allowed to agitate for an additional of 1 day before it was poured slowly into a beaker that contained absolute ethanol so as to precipitate out the product polymer. The product polymer was washed several times with fresh absolute ethanol before drying under vacuum at 160 °C overnight.

### 2.4. Synthesis of 6FDA-TMPDA Polymer

**Figure 2:**
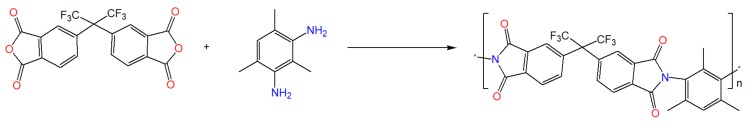
Reaction scheme of 6FDA-TMPDA synthesis.

Separately, the 6FDA-TMPDA polymer (Figure 2) was developed using the method as described below [19]. Similar to ODPA-TMPDA, the whole synthesis was conducted in an inert atmosphere. 6FDA (0.44 g) was first added into a round-bottom flask. NMP (1.6 mL) and TMPDA (0.15 g) was then gradually added. The mixture was stirred for about 30 min before the solution was diluted further with 9.6 mL of NMP. 5-h agitation of the mixture was conducted to ensure that the viscous polyamic acid solution can be formed. This was followed by the imidization process, with the addition of TEA (0.20 g) and Ac_2_O (0.82 g). The process is allowed to go to completion by mixing for 20 h. The solution was finally precipitated with methanol, where the polymer was washed several times with fresh methanol before drying under vacuum at 160 °C overnight.

### 2.5. Membrane Fabrication

The fabrication of dense mixed-matrix membranes was adopted via solution casting approach. First, 0.5 g of nanocrystal HKUST-1 was dispersed in 3 mL chloroform. The potential agglomeration of nanocrystal HKUST-1 was minimized with the addition of sonication horn for 5-min duration (Qsonica, Q125, Newtown, CT, USA). After the sonication completes, the polymers were added into the solution and the resulting mixture was agitated overnight. Next, the dope solution was poured onto a flat glass plate and cast into a continuous film. The thickness of the membrane was controlled with the use of a casting knife. The membranes were cast inside a glove bag to ensure a casting environment filled with chloroform vapor so as to inhibit rapid solvent evaporation. After providing sufficient evaporation time for phase inversion, a vacuum oven was used to anneal the resulting membranes at 160 °C for 24 h.

### 2.6. Characterization

#### 2.6.1. Characterization of Nanocrystal HKUST-1

NOVATouch LX2 (volumetric gas sorption analyzer, Quantachrome, Boynton Beach, FL, USA) was utilized in this study to investigate the C_2_H_4_ and C_2_H_6_ adsorption properties of the nanocrystal HKUST-1. The nanocrystal HKUST-1 was first outgassed at 160 °C for 1 day under high vacuum to remove any potential residual solvents in the nanocrystal HKUST-1. C_2_H_4_ and C_2_H_6_ isotherms were measured in the range of 0–1 bar at 25 and 35 °C. Water circulator is used to ensure that the temperature does not fluctuate during the measurement. The isotherms of C_2_H_4_ and C_2_H_6_ were fitted accordingly with dual-site and single-site Langmuir equations (Equations (1) and (2)) [55].

(1) $$q=\frac{q_{sat,1}b_{1}p}{1+b_{1}p}+\frac{q_{sat,2}b_{2}p}{1+b_{2}p}$$

(2) $$q=\frac{q_{sat}b_{1}p}{1+b_{1}p}$$

where $q_{sat,1}$, $q_{sat,2}$ = saturation loading in mmol g^−1^, $b_{1}$, $b_{2}$ = Langmuir constant in bar^−1^, p = pressure in bar, and q = amount of C_2_H_4_ and C_2_H_6_ adsorption in mmol g^−1^. The mixture–gas selectivity (Ideal Adsorbed Solution Theory, IAST) was used to determine the C_2_H_4_/C_2_H_6_ selectivity of the nanocrystal HKUST-1 [56], as exemplified in Equation (3):

(3) $$Selectivity=\frac{x_{1}/x_{2}}{y_{1}/y_{2}}$$

where $x_{1}$, $x_{2}$ = mole fractions of the adsorbed phase, and $y_{1}$, $y_{2}$ = mole fraction of the gas phase. Isosteric heat of adsorption, $-Q_{st}$ was determined from the Clausius-Clapeyron equation by measuring the C_2_H_4_ and C_2_H_6_ isotherms of nanocrystal HKUST-1 at 25 and 35 °C [57,58,59].

(4) $$-Q_{st}=RT^{2}{\left(\frac{\partial lnp}{\partial T}\right)}_{q}$$

In this expression, R = molar gas constant, T = temperature in K, p = pressure in bar and q = adsorbed amount in mmol g^−1^, which can be obtained either from the dual-site or single-site Langmuir equation. Besides, volumetric gas sorption analyzer was used to measure N_2_ physisorption at −196 °C (77 K) to determine the porosity properties of the nanocrystal HKUST-1, for which similar activation condition as elaborated above was utilized. A D2 phaser (X-ray diffraction, Bruker, Billerica, MA, United States) was used to verify the crystallinity of the nanocrystal HKUST-1 using a laser beam (with a CuKα radiation, 0.154 nm). At room temperature, the analysis condition (step size of 0.02° and 2*θ* from 5 to 40°) was set. Field-emission scanning electron microscope (FESEM, JSM6701, JEOL, Akishima, Japan), which is set at 5 kV acceleration voltage, was used to investigate the structural morphology of nanocrystal HKUST-1. The particle size distribution (mean and standard deviation) of nanocrystal HKUST-1 was calculated with the aid of image analysis tool (Nano Measure). The thermal stability of nanocrystal HKUST-1 was measured with the use of a thermogravimetric analyzer (TGA, SDT Q600, TA instrument, New Castle, DE, United States), which was conducted under a temperature scan of 40 to 800 °C at a ramping rate of 10 °C min^−1^. Nitrogen gas is used as purging gas at a flow rate of 100 mL min^−1^. Change in mass per unit temperature (dm/dT) was calculated to investigate the thermal behavior of nanocrystal HKUST-1.

#### 2.6.2. Characterization of Mixed-Matrix Membranes

FESEM (JSM6701, JEOL, Akishima, Japan) at 5 kV acceleration voltage was used to determine the cross-sectional morphology of the mixed-matrix membranes. The membranes were fractured under liquid nitrogen prior to the gold coating process. Fourier transform-infrared spectroscopy (FT-IR, PerkinElmer, Spectrum One, Waltham, MA, USA) was used to understand the functional groups of the pure polymeric membrane. The measurement was conducted in the range of 4000 to 450 cm^−1^ with a 4 cm^−1^ resolution. TGA (SDT Q600, TA instrument, New Castle, DE, USA) was used to determine the thermal properties of both mixed-matrix and polymeric membranes. Similarly, dm/dT was also calculated to investigate the behavior of membranes developed in this work. Temperature scan (40 to 800 °C) and ramping rate at 10 °C min^−1^ (together with the purging of pure nitrogen at 100 mL min^−1^) were set. An analytical balance with a density kit feature (Mettler Toledo, ME204, Columbus, OH, USA) was utilized to determine the respective density of mixed-matrix and polymeric membranes. This value was calculated by measuring the sample in both auxiliary liquid (ethanol) and air using Archimedes’ principle.

#### 2.6.3. Mixture Gas Permeation Analysis

Mixture gas permeation analysis was measured by using a permeation setup from GTR Tec Corporation (constant pressure-variable volume system). The ethylene/ethane mixture (C_2_H_4_/C_2_H_6_ = 50:50 vol%) and helium gas (He, 99.9995%) utilized in this set-up were obtained from Air Liquide, Singapore Pte Ltd. This composition of the C_2_H_4_/C_2_H_6_ mixture has been widely used as an initial screening for the preliminary investigation of the separation performance of membranes and porous materials [60,61,62]. Hence, we have adopted the same C_2_H_4_/C_2_H_6_ mixture composition in this work. To measure the separation performances, the membrane was first mounted in a permeation cell (permeation area = 1.77 cm^2^). The up and downstream of the membrane were subjected to test gas (C_2_H_4_/C_2_H_6_ mixture) and inert carrier gas (i.e., He), which flow rate is set as 20 sccm and 5 sccm, respectively. Mass flow controllers were used to control the flow rate. At periodic time interval, gases that permeated through the membrane were sampled by a gas chromatography fitted with a thermal conductivity detector (TCD), using He as a sweeping gas. The steady-state reading was taken after the concentrations of C_2_H_4_ and C_2_H_6_ does not demonstrate substantial fluctuation. During the measurement, the environment was remained isothermal (35 °C). At least three different samples for each membrane type were measured to ensure that the gas permeation results are reproducible, with the error bars determined by standard deviation.

#### 2.6.4. C_2_H_4_ and C_2_H_6_ Adsorption Analysis of Membranes

Similarly, pure component C_2_H_4_ and C_2_H_6_ adsorption of each respective membrane was evaluated using the conditions as elaborated in Section 2.6.1 to give the solubility–diffusivity behavior of the membranes. The same activation conditions as described above were used for all membranes. All the isotherms were fitted using single-site Langmuir equation (equation 2) so as to determine the C_2_H_4_ and C_2_H_6_ adsorption at a specified pressure. The results of all parameters were summarized in Table S1. The solubility, S, of C_2_H_4_ and C_2_H_6_ in each membrane can be determined using Equation (5):

(5) $$S=\frac{q\rho }{p}$$

where q = amount of gas adsorbed per unit membrane mass based on a specified pressure, p, and ρ = membrane’s density. The gas diffusivity, D, was calculated by taking the ratio between permeability, P and solubility, S, given that P=DS. The units of P and S are expressed as $mol\cdot {m/m}^{2}\cdot s\cdot \mathrm{bar}$ and ${mol/m}^{3}\cdot bar$, respectively.

## 3. Results and Discussion

### 3.1. Characterization of Nanocrystal HKUST-1

**Figure 3:**
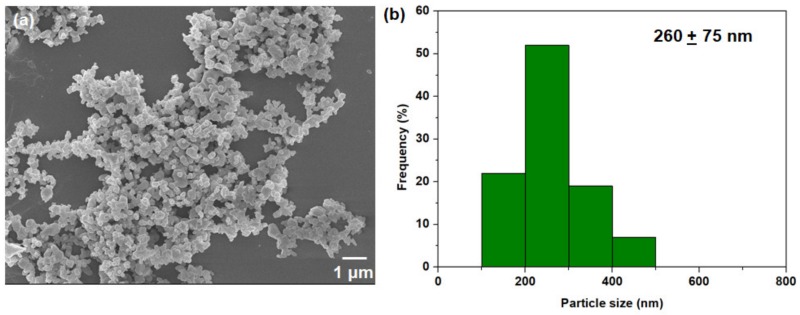
(**a**) FESEM image of nanocrystal HKUST-1; (**b**) Particle size distribution of nanocrystal HKUST-1. The mean and standard deviation are included in the figure.

XRD (X-ray diffraction) as shown in Figure S1a was first verified to confirm the crystallinity of nanocrystal HKUST-1. The diffraction peaks are in general similar to the data reported in the literature [34,63], as well as the simulated pattern of HKUST-1 [64]. N_2_ physisorption measurement at −196 °C (77 K) (Figure S1b) showed a large N_2_ sorption at low P/P_o_, which is a clear indication of a Type 1 isotherm. In other words, large micropore volumes are present in our nanocrystal HKUST-1 sample (Table S2). The FT-IR analysis indicates a successful formation of the characteristic Cu_2_(COO)_4_ paddle wheel of HKUST-1, on the basis of the absorption bands detected at 1647, 1615, 1451 and 1376 cm^−1^ (Figure S1c) [65]. Besides, the nanocrystal HKUST-1 is thermally stable up until 350 °C, as shown by the TGA curve (Figure S1d). Successful nanosizing of HKUST-1 crystals was demonstrated through the FESEM image (Figure 3a). As exemplified by the particle size distribution (Figure 3b), the mean particle size is about 260 nm with a distribution ranging from 100–500 nm, which is a stark contrast to the micron-sized Basolite C300 HKUST-1 bulk crystals [66].

### 3.2. C_2_H_4_ and C_2_H_6_ Adsorption of Nanocrystal HKUST-1

**Figure 4:**
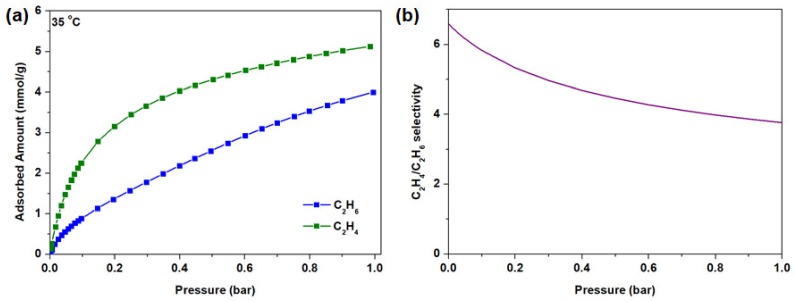
(**a**) C_2_H_4_ and C_2_H_6_ adsorption isotherm of nanocrystal HKUST-1 at 35 °C; (**b**) The Ideal Adsorbed Solution Theory (IAST) selectivity of HKUST-1 nanocrystal at 35 °C as a function of feed pressure.

To attest the merits of using nanocrystal HKUST-1, the C_2_H_4_ and C_2_H_6_ adsorption isotherms were studied at 35 °C (Figure 4a). The C_2_H_4_ and C_2_H_6_ isotherm at 25 °C were also included in Figure S2a,b (fitting parameters are summarized in Table S3) and the results were used in the calculation of the isosteric heat of adsorption ($-Q_{st}$). Generally, nanocrystal HKUST-1 showed a preferential adsorption towards C_2_H_4_and C_2_H_6_ despite the latter having a higher polarizability (C_2_H_4_: 42.5 × 10^−25^ cm^3^ vs. C_2_H_6_: 44.3 × 10^−25^ cm^3^) [67]. We attribute this observation to the presence of coordinatively unsaturated metal sites that allows the formation of olefin (C_2_H_4_) complexation. Metal sites are generally capable of accepting π electron from the olefin as well as donating electrons to the empty π* antibonding orbital of the olefin, thus leading to a stronger C_2_H_4_ interaction by the metal cations, which in this case is Cu^2+^ [68,69]. This is further verified by a higher $-Q_{st}$ at zero coverage for C_2_H_4_ (36.3 kJ mol^−1^) as compared to C_2_H_6_ (27.9 kJ mol^−1^) (Figure S2c), indicating that the adsorption of olefin to the unsaturated metal sites is indeed favorable. The overall IAST selectivity as a function of the feed pressure was plotted in Figure 4b, where the C_2_H_4_/C_2_H_6_ selectivity is approximately at 3.7 under 1 bar feed pressure.

### 3.3. Characterizaiton of Mixed-Matrix Membranes

**Figure 5:**
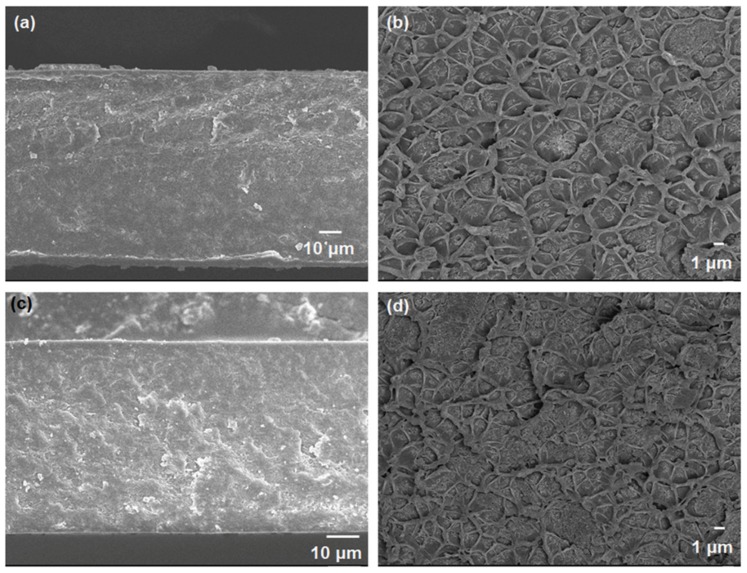

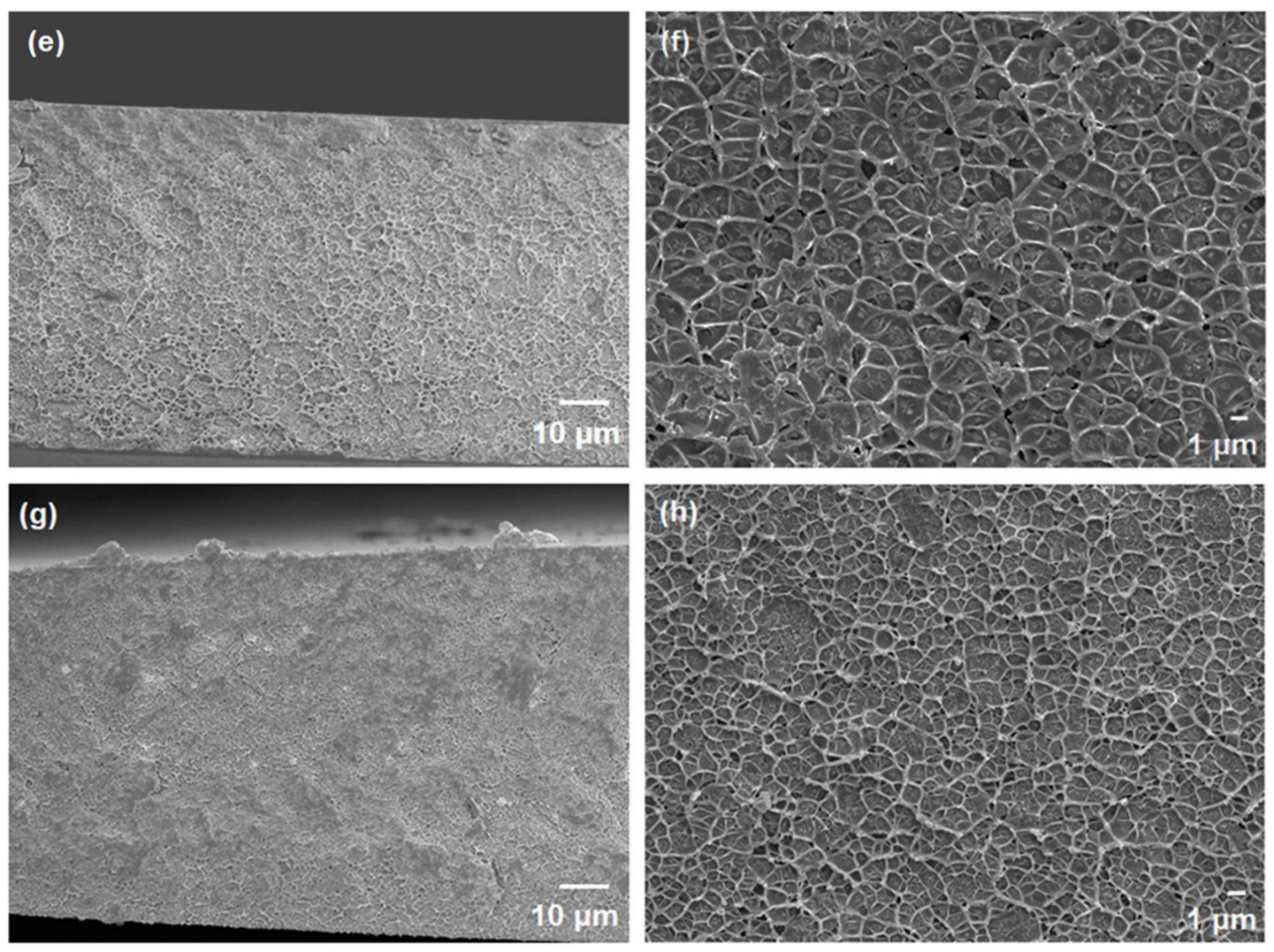
FESEM images of the mixed-matrix membranes: (**a**,**b**) 10 wt%, and (**c**,**d**) 20 wt% HKUST-1 with ODPA-TMPDA; and (**e**,**f**) 10 wt%, and (**g**,**h**) 20 wt% HKUST-1 with 6FDA-TMPDA.

In this work, in-house polyimide ODPA-TMPDA and 6FDA-TMPDA polymers were used for mixed-matrix membrane fabrication. To ensure successful syntheses of the polyimides, FT-IR spectroscopy was conducted to verify the functional groups (Figure S3). In general, both polymers showed the characteristic imide peaks at 1774 cm^−1^ and 1719 cm^−1^ that corresponded to asymmetric and symmetric C=O stretching, respectively. Besides, C-N stretching (1358 cm^−1^) was also detected in both spectra [39,70,71]. As the polyimides were synthesized via a two-step reaction (where monomers were first condensed to form polyamic acid before subsequent chemical imidization), it is important to determine that all polyamic acid has been successfully reacted. This is evidenced by the absence of the O-H peak (3500 cm^−1^), which indicates a successful imidization process (Figure S3). The resulting polymer is well-dissolved in chloroform, which is a typical solvent used in polymeric membrane fabrication. Hence, mixed-matrix membranes were developed from these polyimides using 10 and 20 wt% loadings of HKUST-1. Typically, a 20 wt% filler loading is not uncommon for a three-dimensional porous filler in a mixed-matrix membrane [19]. In this work, the 20 wt% loading of HKUST-1 is the optimal amount, as also evidenced by other studies on mixed-matrix membranes [72,73]. Next, the structural morphology of the membranes was examined using FESEM (Figure 5). In general, the cross-sectional morphology of all membranes exhibits an intact and homogeneous integrity. The polymer/filler interface is visibly free from the sieve-in-a-cage morphology—a defective interface that is commonly found in zeolite-based mixed-matrix membranes [74]. This good structural integrity stems from the organic moieties of the nanocrystal HKUST-1, which increases the compatibility between the filler and polymer. The nanosized HKUST-1 crystals can also facilitate easy wrapping by the polyimide chains, and coupled with the intrinsically good compatibility, a more intimate contact at the polymer/filler interface can be realized, rendering a defect-free mixed-matrix membrane [50,66]. Furthermore, as compared with micron-sized bulk HKUST-1 crystals, our nanosized HKUST-1 are less prone to particle sedimentation during membrane fabrication. This helps create a uniform filler dispersion as well as homogenous mixed-matrix membrane morphology, as evidenced by our FESEM images (Figure 5). In contrast, as observed by several other studies, micron-sized fillers are more likely to settle at the bottom of the membrane, creating potential defects in the membrane structure [19,75]. Additionally, TGA analysis revealed that the thermal stability of the polymers remained unchanged with the incorporation of nanocrystal HKUST-1 in the mixed-matrix membranes (Figure S4). Based on the profile, it can be observed that there are two peaks in the case of mixed-matrix membrane as compared with the one peak in pure polymeric membrane. The first peak corresponds to the degradation of the HKUST-1 nanocrystals in the membrane, as corroborated by the thermal stability profile of the nanocrystal HKUST-1 (Figure S1d). The second peak is attributed to the degradation of the polymer matrices. In terms of the mechanical properties of the mixed-matrix membranes, we are expecting a marginal decrease in comparison to the pure polymeric membranes due to the incorporation of the nanocrystal HKUST-1. However, this anticipated drop is unlikely to create a negative impact as the membranes continued to demonstrate good manageability and robustness during measurements and characterization. As reported by Ge and coworkers, the problem only surfaced at 50 wt% HKUST-1 loading when the mechanical properties of the membranes were reportedly found to be insufficient to support normal performance measurements [76].

### 3.4. Gas Permeation Behavior of Membranes

**Figure 6:**
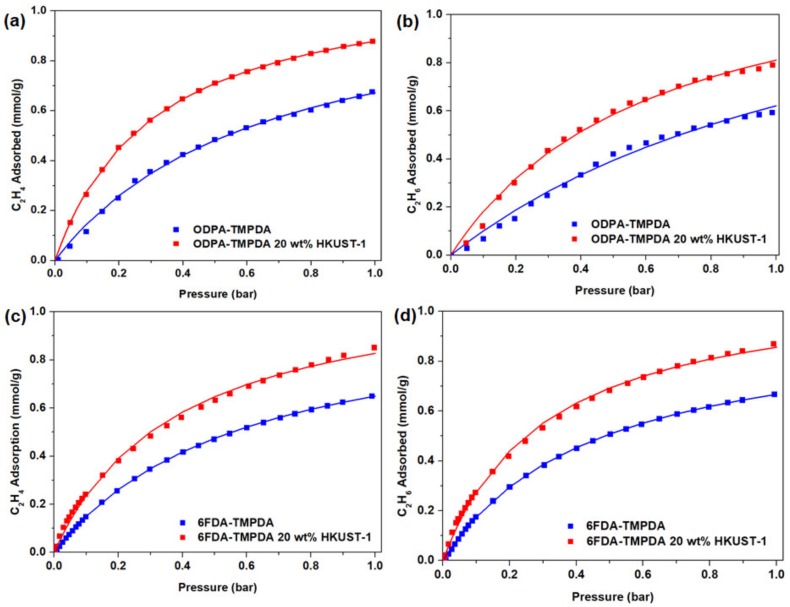
Adsorption isotherm of (**a**,**c**) C_2_H_4_ and (**b**,**d**) C_2_H_6_ of pure polymeric and mixed-matrix membranes.

Gas permeation analysis of each membrane was performed at 35 °C under 1 bar upstream pressure using ethylene/ethane (C_2_H_4_/C_2_H_6_) gas at 50/50 mixture, with results summarized in Table 1. In general, a substantial enhancement in the C_2_H_4_ permeability was observed when HKUST-1 was loaded. Particularly, the incorporation of 20 wt% HKUST-1 gave a 155% and 69% improvement in C_2_H_4_ permeability when ODPA-TMPDA and 6FDA-TMPDA was used as the matrix, respectively. The improvement in permeability was, however, accompanied by a marginal drop in the C_2_H_4_/C_2_H_6_ selectivity. We reckon that the large pore window in the nanocrystal HKUST-1 reduces the transport resistance and allows faster diffusion, but it was not selective. Next, to attest our claims, we quantify the C_2_H_4_ and C_2_H_6_ diffusivity and solubility of these membranes (Figure 6). The calculated C_2_H_4_ and C_2_H_6_ diffusivity and solubility are compiled accordingly (Table 2). Based on the isotherm profile, substantially higher C_2_H_4_ and C_2_H_6_ adsorptions were observed for the mixed-matrix membranes as compared to pure polymeric membranes. This increases both the absolute values of the C_2_H_4_ and C_2_H_6_ diffusivity and solubility of the mixed-matrix membranes. Nevertheless, the solubility selectivity remains relatively constant while the diffusivity selectivity decreases slightly. Evidently, our results suggest that the incorporation of nanocrystal HKUST-1 indeed provides diffusion pathways of lower resistance to facilitate gas transport, but these pathways are indiscriminatory towards C_2_H_4_ and C_2_H_6_. The selectivity continues to be delivered by the polyimide matrices.

### 3.5. Comparison of Gas Separation Performance

**Figure 7:**
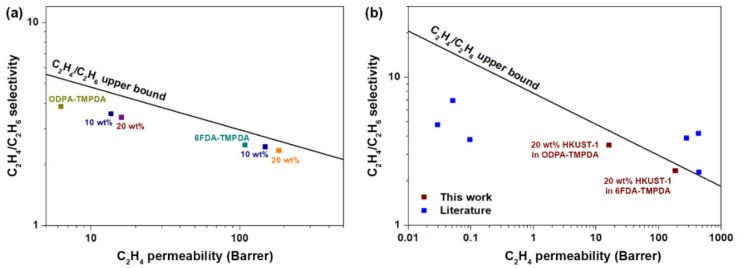
(**a**) Comparison of the synthesized membranes (pure polymer and mixed-matrix) with reference to the C_2_H_4_/C_2_H_6_ upper bound. The upper bound is constructed based on ref. [24]. (**b**) Mixture–gas performance comparison of our mixed-matrix membranes with literature data [52,60].

Subsequently, our mixed-matrix membranes were benchmarked against an upper bound for the C_2_H_4_/C_2_H_6_ gas separation performance. Besides, the membrane performances were compared to the current literature data. As demonstrated in Figure 7a, the performances of our mixed-matrix membranes edge generally close to the upper bound limit as compared to the pure polymeric membranes. The choice of polymer matrix is an important parameter that can critically affect the overall performance of the mixed-matrix membranes. This deduction is corroborated as we compare the performance of our mixed-matrix membranes with the current literature data. Furthermore, most studies to date mainly focus on pure gas permeation (Table 3), giving only ideal C_2_H_4_/C_2_H_6_ selectivity. While evaluating ideal selectivity is a convenient way to assess membrane performance, it is not entirely representative as we know that competitive sorption by two gases of similar polarizabilities and dipole moments can result in lower than expected performance, rendering most results in the literature overrated. Thus, to indicate the potential relevance in industrial gas separation process, the investigation of gas permeation under mixture–gas is always desirable as mixture–gas evaluation generally gives more accurate gas separation performances. Comparing mixture–gas performances with others in the literature [52,60], our mixed-matrix membranes are highly competitive, as shown in Figure 7b. There are some high-performing mixed-matrix membranes, comprising advanced MOFs (MOF-74) [60], which exhibit separation performances that transcend the upper bound limit (Figure 7b). From a practical perspective, such tailor-made MOFs are not readily scalable and have a lower potential for commercialization. In contrast, commercial HKUST-1 (Basolite C300) is already available in the market, and hence up-scaling nanocrystal HKUST-1 is deemed less challenging. On this basis, we believe that leveraging nanocrystal HKUST-1 as a filler for mixed-matrix membranes for C_2_H_4_/C_2_H_6_ separation is not only effective but also attractive.

## 4. Conclusions

Nanocrystal HKUST-1 was successfully synthesized and incorporated as mixed-matrix membranes in both ODPA-TMPDA and 6FDA-TMPDA matrices. Nanosizing HKUST-1 not only helps to mitigate polymer/filler interfacial defects, but also achieves a homogeneous morphology of the mixed-matrix membranes. The coordinatively unsaturated open metal sites of the nanocrystal HKUST-1 favor C_2_H_4_ adsorption. At 20 wt% nanocrystal HKUST-1 loading, the C_2_H_4_ permeability was found to increase up to 155% with a marginal drop in the C_2_H_4_/C_2_H_6_ selectivity. We attribute this to the large pore size of HKUST-1 (9 × 9 Å), which provides indiscriminatory diffusion pathways of lower resistance to the gas molecules. Solubility–diffusivity analysis of both C_2_H_4_ and C_2_H_6_ gases corroborated this conclusion. In addition, it was revealed that the polymer matrices continued to deliver the required C_2_H_4_/C_2_H_6_ selectivity as demonstrated by the relatively constant solubility selectivity in the mixed-matrix membranes. As a result, the performance of our mixed-matrix membranes was found to edge closer towards the upper bound, enabling nanocrystal HKUST-1 as an effective porous filler to enhance the performance of mixed-matrix membranes for ethylene/ethane separation.

## Figures and Tables

**Table 1:** C_2_H_4_/C_2_H_6_ permeation behavior of pure ODPA-TMPDA and 6FDA-TMPDA membranes together with nanocrystal HKUST-1-containing mixed-matrix membranes. ^a^

Membrane	C_2_H_4_ Permeability (Barrer)	C_2_H_4_/C_2_H_6_ Selectivity
ODPA-TMPDA	6.3 ± 0.4	3.9 ± 0.4
ODPA-TMPDA + 10 wt% HKUST-1	13.6 ± 1.7	3.6 ± 0.3
ODPA-TMPDA + 20 wt% HKUST-1	16.0 ± 0.2	3.4 ± 0.2
6FDA-TMPDA	108 ± 7.2	2.5 ± 0.1
6FDA-TMPDA + 10 wt% HKUST-1	148 ± 11.8	2.5 ± 0.5
6FDA-TMPDA + 20 wt% HKUST-1	183 ± 3.8	2.4 ± 0.1

^a^ The measurement condition is stated as follows: 1 bar feed pressure and C_2_H_4_/C_2_H_6_ mixture (50/50) at 35 °C.

**Table 2:** C_2_H_4_ and C_2_H_6_ solubility and diffusivity parameters of pure polymeric and mixed-matrix membranes at 35 °C.

Membrane	Density (g cm^−3^)	C_2_H_4_ Solubility, × 10^3^ (mol m^−3^ bar^−1^)	C_2_H_4_ Diffusivity, × 10^−13^ (m^2^ s^−1^)	C_2_H_6_ Solubility, × 10^3^ (mol m^−3^ bar^−1^)	C_2_H_6_ Diffusivity, × 10^−13^ (m^2^ s^−1^)	Solubility Selectivity	Diffusivity Selectivity
ODPA-TMPDA	1.25	1.21	0.394	0.989	0.124	1.22	3.17
ODPA-TMPDA + 20 wt% HKUST-1	1.24	1.77	0.688	1.46	0.244	1.21	2.82
6FDA-TMPDA	1.26	1.19	0.687	1.28	2.57	0.933	2.68
6FDA-TMPDA + 20 wt% HKUST-1	1.22	1.58	0.880	1.69	3.50	0.934	2.51

**Table 3:** Summary of C_2_H_4_/C_2_H_6_ pure component gas permeation results of the mixed-matrix membranes that are available in the literature. **^a^**

Filler	Polymer	Filler Loading (wt%)	Separation Performance						Ref.
			Testing Condition		*P*(C_2_H_4_) (Barrer)	Permeability Enhancement (%)	α(C_2_H_4_/C_2_H_6_)	Selectivity Enhancement (%)	
			Pressure (bar)	Temp. (°C)					
Silica nanoparticles	CA	30	2	35	0.11	100	4.1	173	[5]
Silica nanoparticles	Matrimid^®^	20	3	30	0.19	137.5	3.2	98.1	[51]
ZIF-8	6FDA-DAM	23.8	2	35	72.9	85.0	3.2	−3.1	[77]
ZIF-8	DBzPBI-BuI	30	2.7	35	111	2953	2.6	−2.29	[13]
HKUST-1 ^b^	ODPA-TMPDA	20	1	35	16	155.2	3.4	−11.6	This work
HKUST-1 ^b^	6FDA-TMPDA	20	1	35	183	69.4	2.4	−6.0	This work

**^a^** Optimal performance in terms of C_2_H_4_ permeability and (or) C_2_H_4_/C_2_H_6_ selectivity were selected and tabulated in this table; **^b^** The data for this work is included as reference.

## Supplementary Materials

The following are available online at https://www.mdpi.com/2077-0375/10/4/74/s1, Figure S1: Characterization of nanocrystal HKUST-1, showing (a) X-ray diffraction pattern, (b) N_2_ physisorption isotherm at 77 K (open and closed symbols indicate adsorption and desorption branches, respectively), (c) FT-IR spectrum, as well as (d) TGA curve, showing weight loss and dm/dT against temperature, of nanocrystal HKUST-1 from 40 to 800 °C. Figure S2: C_2_H_4_ and C_2_H_6_ adsorption of HKUST-1 at (a) 25 °C and (b) 35 °C, (c) −*Q*_st_ of HKUST-1 for C_2_H_4_ and C_2_H_6_. Figure S3: FT-IR spectrum of (a) ODPA-TMPDA and (b) 6FDA-TMPDA polymer. Figure S4: TGA analysis (a,b) weight loss against temperature and (c,d) dm/dT against temperature of 10 wt% and 20 wt% nanocrystal HKUST-1 in (a,c) ODPA-TMPDA and (b,d) 6FDA-TMPDA polymer. Table S1: Fitting parameters for C_2_H_4_ and C_2_H_6_ for membranes at 35 °C. Table S2: Porosity properties of nanocrystal HKUST-1 based on N_2_ physisorption at 77 K. Table S3: Fitting parameters for C_2_H_4_ and C_2_H_6_ for HKUST-1 at 25 and 35 °C.

## Nomenclature

List of symbols	
$b_{1}$, $b_{2}$	Langmuir constant ($bar^{-1}$)
dm/dT	Change in mass per unit temperature ($mg\;{}^{o}C^{-1}$)
D	Diffusivity ($m^{2}/s$)
p	Pressure (bar)
P	Permeability (Barrer or $mol\cdot m/m^{2}\cdot s\cdot bar$)
$-Q_{st}$	Isosteric heat of adsorption ($kJ\;mol^{-1}$)
q	Adsorbed amount (mmol g^−1^)
$q_{sat,1}$, $q_{sat,2}$	Saturation loading (mmol g^−1^)
R	Molar gas constant ($J\;mol^{-1}\;K^{-1}$)
S	Solubility ($mol/m^{3}\cdot bar$)
T	Temperature (K)
$x_{1}$, $x_{2}$	Mole fractions of the adsorbed phase
$y_{1}$, $y_{2}$	Mole fractions of the gas phase
ρ	Density ($g\;cm^{-3}$)
Abbreviations	
6FDA	4,4′-(hexafluoroisopropylidene) diphthalic anhydride
Ac_2_O	Acetic anhydride
BuI	5-*tert*-butyl isophthalic acid
C_2_H_4_	Ethylene
C_2_H_6_	Ethane
CA	Cellulose acetate
Cu(NO_3_)_2_.3H_2_O	Copper(II) nitrate trihydrate
C_9_H_6_O_6_	Trimesic acid
DAM (or TMPDA)	2,4,6-trimethyl-*m*-phenylenediamine
DBzPBI	Substituted polybenzimidazole
DMAc	Dimethylacetamide
NMP	N-methyl-2-pyrrolidone
ODPA	4,4′-oxydiphthalic anhydride
PBI	Polybenzimidazole
TEA	Triethylamine
