# Supplementary material for: Leveraging Nanocrystal HKUST-1 in Mixed-Matrix Membranes for Ethylene/Ethane Separation
Source: Membranes (Basel). 2020 Apr 16;10(4):74. doi: 10.3390/membranes10040074 (PMC7231397; doi:10.3390/membranes10040074)
Supplement: Supplementary file 1 [file membranes-10-00074-s001.pdf]

Supplementary Materials

for

Leveraging Nanocrystal HKUST-1 in Mixed-matrix Membranes for Ethylene/Ethane Separation

Chong Yang Chuah [1], S.A.S.C. Samarasinghe [1], Wen Li [2], Kunli Goh [1,*] and Tae-Hyun Bae [3,*]

[1] Singapore Membrane Technology Centre, Nanyang Environment and Water Research Institute, Nanyang Technological University, Singapore 637141, Singapore; chongyang.chuah@ntu.edu.sg (C.Y.C); samarasi001@e.ntu.edu.sg (S.A.S.C.S.); gohkunli@ntu.edu.sg (K.G.)

[2] School of Chemical and Biomedical Engineering, Nanyang Technological University, Singapore 637459, Singapore; liwe0025@e.ntu.edu.sg (W.L.)

[3] Department of Chemical and Biomolecular Engineering, Korea Advanced Institute of Science and Technology, Daejeon, 34141, Republic of Korea; thbae@kaist.ac.kr (T-H.B.)

* Correspondence: gohkunli@ntu.edu.sg; thbae@kaist.ac.kr

Table S1. Fitting parameters for C_2H_4 and C_2H_6 for membranes at 35 °C.

Unit of p = bar; Unit of q = mmol g^{-1}

$$q = \frac{q_{sat} b_1 p}{1 + b_1 p}$$

Sample	*gas*	$q_{sat,1}$	b_1	R^2 value
ODPA-TMPDA	C_2H_4	1.111	1.533	0.9974
	C_2H_6	1.459	0.7410	0.9896
ODPA-TMPDA + 20 wt% HKUST-1	C_2H_4	1.153	3.207	0.9998
	C_2H_6	1.318	1.599	0.9928
6FDA-TMPDA	C_2H_4	1.032	1.698	1
	C_2H_6	0.9732	2.181	0.9999
6FDA-TMPDA + 20 wt% HKUST-1	C_2H_4	1.147	2.588	0.9964
	C_2H_6	1.121	3.231	0.9978

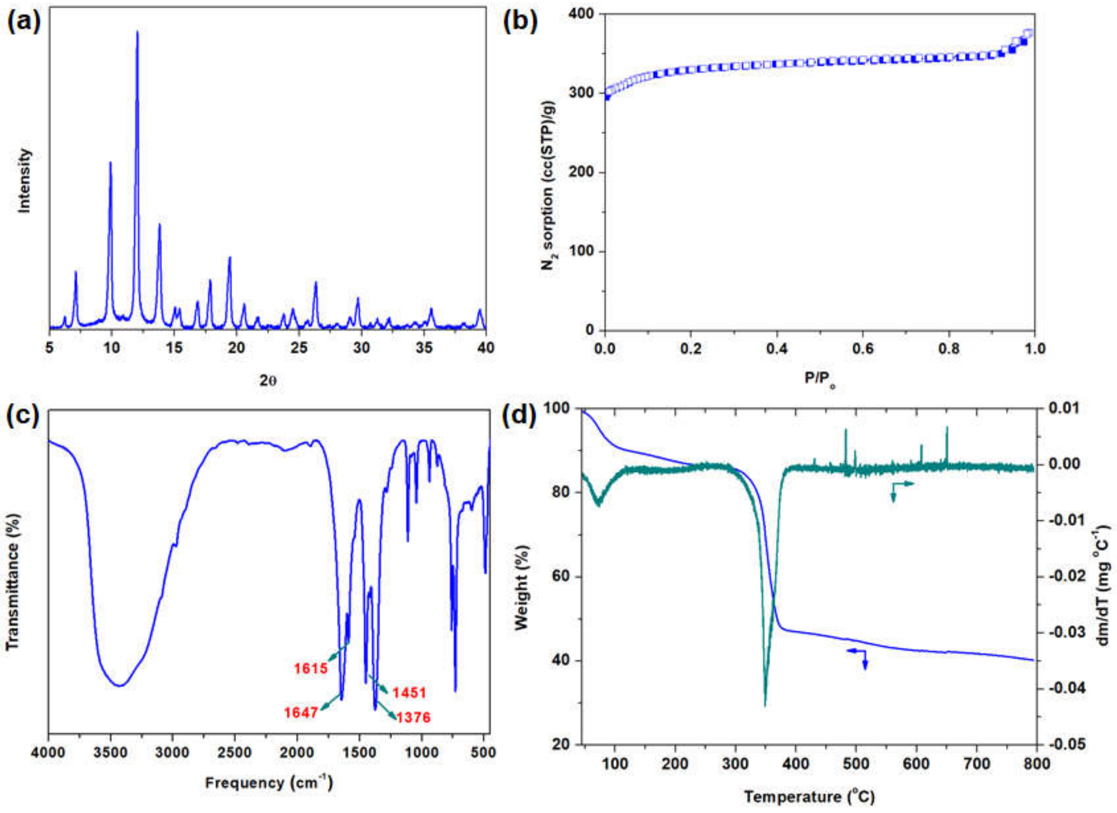

Figure S1. Characterization of nanocrystal HKUST-1, showing **(a)** X-ray diffraction pattern; **(b)** N_2 physisorption isotherm at 77 K (open and closed symbols indicate adsorption and desorption branches, respectively); **(c)** FT-IR spectrum, as well as **(d)** TGA curves, showing weight loss and dm/dT against temperature, of nanocrystal HKUST-1 from 40 to 800 °C.

Table S2. Porosity properties of nanocrystal HKUST-1 based on N_2 physisorption at 77 K.

S_{BET}[a] (m^2/g)	S_{LANG}[a] (m^2/g)	S_{micro}[b] (m^2/g)	V_{micro}[b] (cc/g)	V_{total}[c] (cc.g)
1125	1464	1087	0.502	0.585

Note: [a]P/P_o range from 0.05 to 0.2 was used to determine the BET and Langmuir surface area; [b]t-plot method was used to calculate the micropore surface area and volume, by choosing the P/P_o range from 0.4 to 0.6; [c]P/P_o = 0.99 was used to calculate the total pore volume.

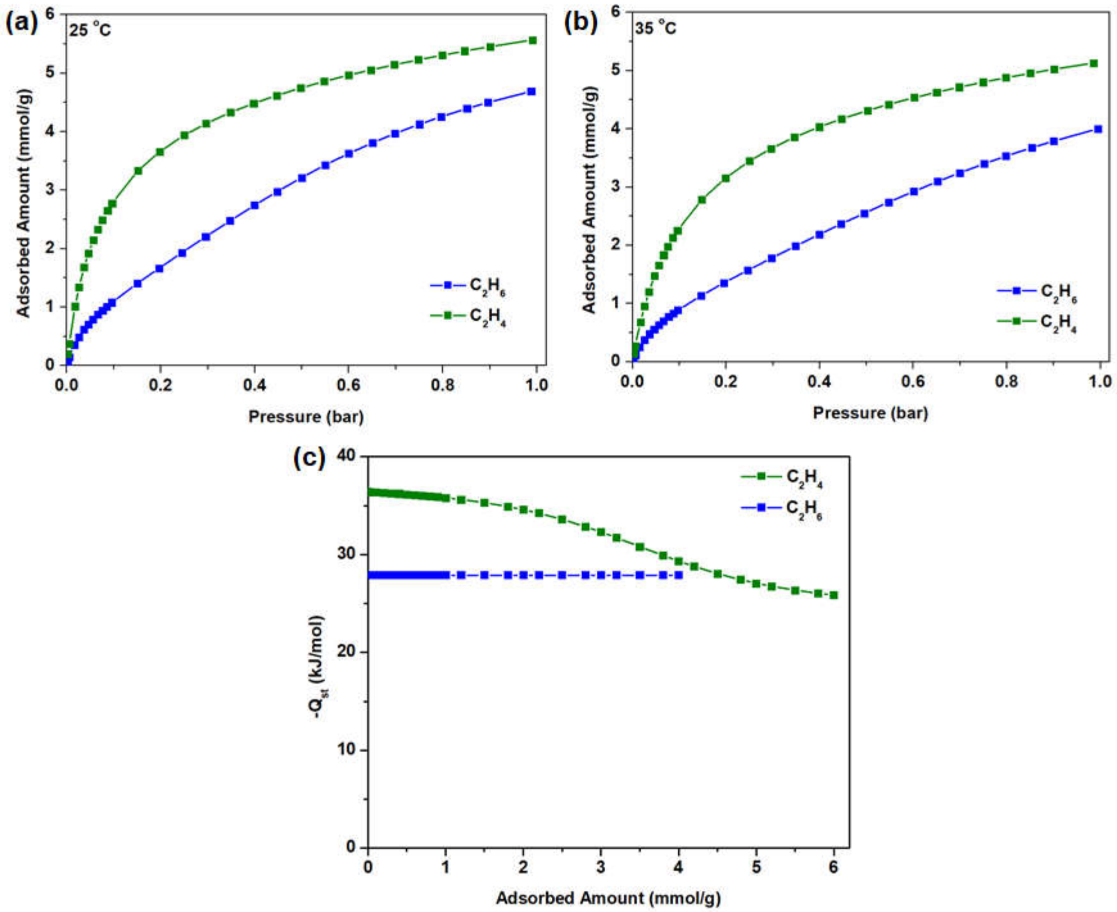

Figure S2. C_2H_4 and C_2H_6 adsorption of HKUST-1 at **(a)** 25 °C and **(b)** 35 °C; **(c)** $-Q_{st}$ of HKUST-1 for C_2H_4 and C_2H_6.

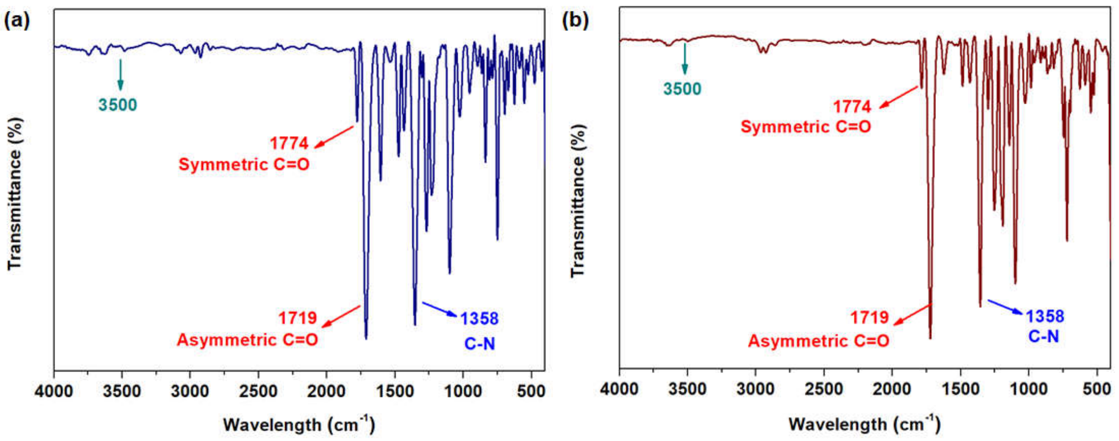

Figure S3. FT-IR spectrum of **(a)** ODPA-TMPDA and **(b)** 6FDA-TMPDA polymer.

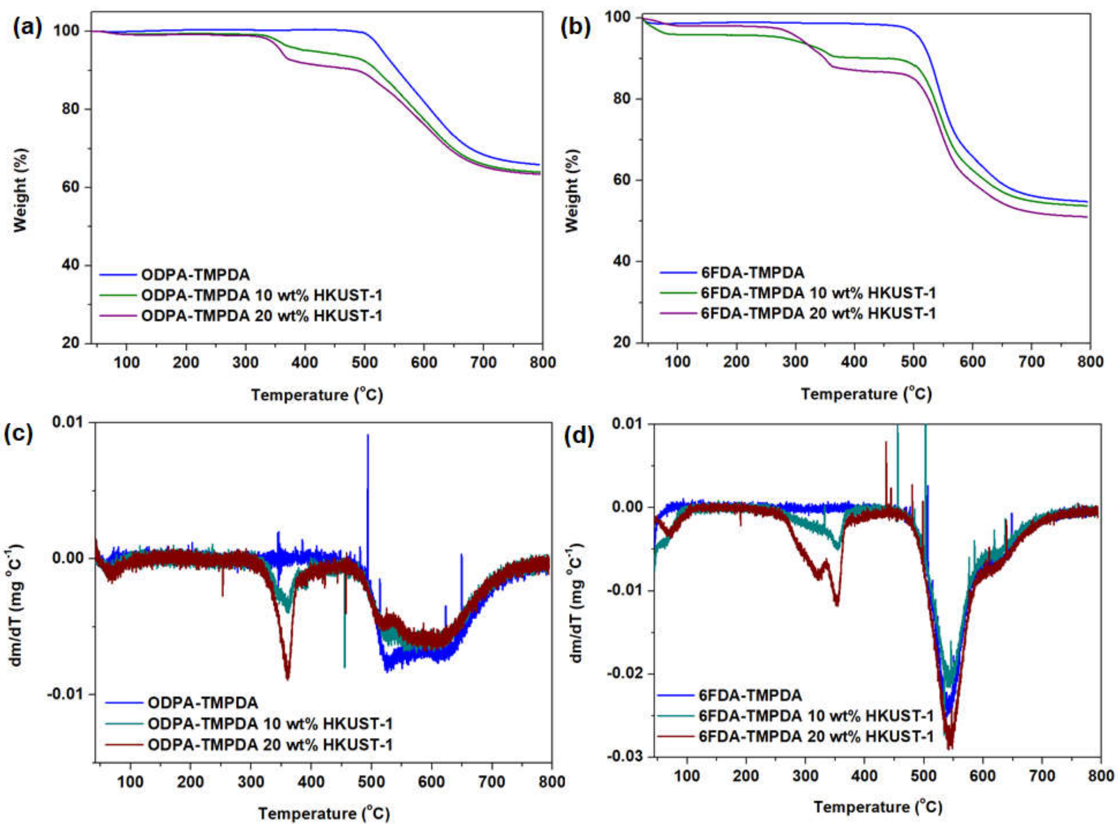

Figure S4. TGA analysis **(a, b)** weight loss against temperature and **(c, d)** dm/dT against temperature of 10 wt% and 20 wt% nanocrystal HKUST-1 loading in **(a, c)** ODPA-TMPDA and **(b, d)** 6FDA-TMPDA polymer.

Table S3. Fitting parameters for C_2H_4 and C_2H_6 for HKUST-1 at 25 and 35 °C.

Unit of p = bar; Unit of q = mmol g^{-1}

$$q = \frac{q_{sat,1} b_1 p}{1 + b_1 p} + \frac{q_{sat,2} b_2 p}{1 + b_2 p}$$

Sample	*gas*	$q_{sat,1}$	b_1	$q_{sat,2}$	b_2	R^2 value
HKUST-1 (25 °C)	C_2H_4	3.563	1.574	3.498	20.29	0.9998
	C_2H_6	7.208	1.697	-	-	0.9921
HKUST-1 (35 °C)	C_2H_4	3.563	1.145	3.498	12.44	1
	C_2H_6	7.208	1.178	-	-	0.9921
